# Characterization of morphology and resistance to *Blumeria graminis* of winter triticale monosomic addition lines with chromosome 2D of *Aegilopstauschii*

**DOI:** 10.1007/s00299-016-2023-x

**Published:** 2016-07-12

**Authors:** M. Majka, M. Kwiatek, J. Belter, H. Wiśniewska

**Affiliations:** Institute of Plant Genetics, Polish Academy of Sciences, Strzeszyńska 34, 60-479 Poznań, Poland

**Keywords:** *Aegilops*, In situ hybridization, Monosomic alien addition lines, Plant height, Powdery mildew, Triticale

## Abstract

*****Key message***:**

**Allocation of the chromosome 2D of*Ae***. ***tauschii*in triticale background resulted in changes of its organization, what is related to varied expression of genes determining agronomically important traits.**

**Abstract:**

Monosomic alien addition lines (MAALs) are crucial for transfer of genes from wild relatives into cultivated varieties. This kind of genetic stocks is used for physical mapping of specific chromosomes and analyzing alien genes expression. The main aim of our study is to improve hexaploid triticale by transferring D-genome chromatin from *Aegilops tauschii* × *Secale cereale* (2*n* = 4*x* = 28, DDRR). In this paper, we demonstrate the molecular cytogenetics analysis and SSR markers screening combined with phenotype analysis and evaluation of powdery mildew infection of triticale monosomic addition lines carrying chromosome 2D of *Ae. tauschii*. We confirmed the inheritance of chromosome 2D from the BC_2_F_4_ to the BC_2_F_6_ generation of triticale hybrids. Moreover, we unveiled a high variable region on the short arm of chromosome 2D, where chromosome rearrangements were mapped. These events had direct influence on plant height of hybrids what might be connected with changes at *Rht8* loci. We obtained 20 semi-dwarf plants of BC_2_F_6_ generation carrying 2D chromosome with the powdery mildew resistance, without changes in spike morphology, which can be used in the triticale breeding programs.

## Introduction

The world production of hexaploid triticale (×*Triticosecale* Wittm.) is rising from 473 thousand tonnes in 2012 to 820 thousand tonnes in 2014 (FAOSTAT 2015). The worldwide expansion of triticale exposed the crop to a variety of stressful environmental conditions (Arseniuk and Góral 2015). From the other side, the global market requires diversified forms of triticale considering grain quality, resistance against biotic and abiotic stresses, and plant morphology. Considering triticale breeding, one of the main goals is to transfer genes of interest from wild relatives into cultivated varieties. The ability of triticale to be crossed with related species allows the addition of the whole genomes or individual alien chromosomes to the triticale complement. Monosomic alien addition lines (MAALs) are genotypes with an alien chromosome from a donor species added to the genome of recipient species. This kind of genetic stocks is widely used for physical mapping of specific chromosomes (Kynast et al. 2004) and analyzing alien genes expression (Cho et al. 2006). The production of alien addition lines and the introgressions with alien chromosome segments carrying target traits can be induced by homoeologous recombination or the gametocidal effect (Kwiatek et al. 2016a, b).

*Ae. tauschii* is a wild, diploid goatgrass (donor of D-genome to bread wheat), characterized by wide genetic variation and close related to the species of the Triticeae tribe. Because of this relationship and variety of genes, this species has been exploited by various groups around the world especially for wheat improvement (Ogbonnaya et al. 2005). The chromosome 2D of *Ae. tauschii* is bearing two important agronomically genes *Rht8* and *Pm43* as well as bh-D1 a multirow spike recessive allele (alias mrs1) determining the supernumerary spikelets trait (Jia et al. 2013). The variation of genes on one chromosome indicates that it is the most valuable one for breeding improvement of Triticeae species.

In triticale breeding programs, reduction of plant height may affect to a better partitioning of assimilates for the benefit of the spike and reducing the risk of lodging what leads to increasing of grain yield (Foulkes et al. 2011). Recently, the height of the cereal plants can be changed by breeders through the use of dwarfing or semi-dwarfing genes. The phenomenon of introduction of the reduced height (*Rht*) genes into bread wheat (*Triticum aestivum* L.) was the major event of ‘green revolution’ (Hedden 2003) and is widely used in wheat breeding. Particulary, the modest height-reducing gene *Rht8* may be suitable to reduce the height of mature plants without compromising early plant growth. The genetic linkage maps presented by Korzun et al. (1998) place the wheat microsatellite marker WMS 261 (*Xgwm261* in map of Somers et al. 2004) 0.6 cM distal to *Rht8* on the short arm of chromosome 2D.

The most important trait for breeding is yield. One of the factors determining this agronomic feature is the amount of grain per plant, which indicates the productiveness of given genotypes. It especially depends on the architecture of the inflorescences. The number of spikelets per rachis node is a key taxonomic trait of the Triticeae tribe. The wheat and rye spike normally bears one spikelet per rachis node, and the formation of supernumerary spikelets (SS) is rare. The loci responsible for the ‘multirow spike’ or MRS trait in wheat and the ‘monstrosum spike’ trait in rye are under the control of a recessive allele at a single locus. The Mrs1 locus is located on chromosome 2DS and the Mo1 locus on chromosome 2RS. Furthermore, there is also identified a homologous loci on the chromosome 2AS of hexaploid, tetraploid, and diploid wheats.

In the last few years, the cultivation of triticale was limited due to the infection by *Blumeria graminis* f. sp. *tritici* which caused powdery mildew (*Pm*) (Czembor et al. 2013). Therefore, resistant varieties are the most feasible means of controlling the disease and reducing yield losses. To date, 54 *Pm* resistance genes where transferred from the wild relatives to wheat (Zhan et al. 2014). However, in the case of triticale, only one work reports the positive transfer of *Pm13* gene from *Aegilopsvariabilis* (Kwiatek et al. 2016a). *Ae*. *tauschii* genomes constitute a great source of *Pm* resistance because of the occurrence of *Pm2*, *Pm34*, and *Pm35* genes on chromosome 5D and *Pm19* on chromosome 7D (Genesymbol, McIntosh et al. 2003). Furthermore, Jia et al. (2013) mapped *Pm43* gene in *Ae*. *tauschii* chromosome 2D, in which the origin source has heretofore been *Th*. *intermedium*. So far, *Th*. *intermedium* resistance gene (later called *Pm43*) was recently found in partial amphiploids with wheat, in the substitution line 2 J(2D), in which a J-chromosome of *Th*. *intermedium* was substituted for chromosome 2D in wheat (Liu and Wang 2005; Liu et al. 2005) as well as transfer to wheat using a resistant partial amphiploid as a bridging parent in crosses with susceptible wheat lines.

Our main goal was to produce triticale MAALs carrying 2D chromosome of *Ae. tauschii*. For that reason, we have introduced D-genome chromosomes into triticale using *Aegilops tauschii* Coss. (DD, 2*n* = 2*x* = 14) × *S. cereale* (RR, 2*n* = 2*x* = 14) amphiploid forms to hybridize with triticale cv. Bogo (Kwiatek et al. 2015). The present study aimed: (1) to characterize the chromosome composition of the BC_2_F_4_ to BC_2_F_6_ generations of *Ae. tauschii* × triticale hybrids; (2) to verify the D-genome composition with selected SSR markers in hybrid plants, and (3) to evaluate the influence of the D-genome introgression onto important agronomic traits, including plant height, spike morphology, and resistance to powdery mildew in comparison with winter triticale cultivar Bogo.

## Materials and methods

### Plant material

Glasshouse experiments were carried out in three subsequent vegetation seasons at the Institute of Plant Genetics, Polish Academy of Sciences in Poznań, Poland. Seeds of *Aegilops tauschii* Coss. (D51; 2*n* = 2*x* = 14; DD), *S*. *cereale* (Strzękęcińskie; 2*n* = 2*x* = 14; RR) and ×*Triticosecale* Wittm. (Bogo; 2*n* = 6*x* = 42; AABBRR) originating from the collection of the Institute of Plant Genetics. The *Ae. tauschii* × *S. cereale* amphiploids (2*n* = 4*x* = 28; DDRR) were obtained using embryo rescue by Sulinowski and Wojciechowska of the Institute of Plant Genetics (data unpublished). The F_1_ (*Ae*. *tauschii* × *S*. *cereale*) × triticale hybrids were obtained by crossing of triticale cv. Bogo with *Ae*. *tauschii* × *S*. *cereale* amphiploids as a pollinator. Backcrosses with the triticale as a male parent were used to achieve following generations (BC_1_F_1_ and BC_2_F_1_) and further self-crossed to produce following generations of BC_2_F_2_ to BC_2_F_6_ hybrid plants. Seeds of *T*. *aestivum* cv. Chinese Spring were kindly supplied by the National Small Grains Collection (USDA-ARS).

### Probe labeling

Total genomic DNA was extracted from fresh leaves of *Ae*. *tauschii* (DD), *Triticum monococcum* (A^m^A^m^), *Ae*. *speltoides* (BB), *S*. *cereale* (RR), and triticale ‘Bogo’ (AABBRR) using GeneMATRIX Plant and Funghi DNA Purification Kit (EURx Ltd.). Genomic DNA from *Ae. tauschii* and *T*. *monococcum* were labeled by nick translation (using NickTranslation Kit, Roche, Mannheim, Germany) with tetramethyl-5-dUTP-rhodamine (Roche) or digoxigenin-11-dUTP (Roche) depending on the visualization concept. Blocking DNA from triticale, *Ae*. *speltoides* and *S*. *cereale*, was sheared to fragments of 5–10 kb by boiling for 30–45 min and used at a ratio of 1:50 (probe:block). The 5S rDNA and 25S rDNA as well as pSc119.2 and pAs1 probes were obtained as have been described by Kwiatek et al. (2016a, b) and labeled with tetramethyl-rhodamine-5-dUTP (Roche), digoxigenin-11-dUTP (Roche), digoxigenin-11-dUTP (Roche), and tetramethyl-rhodamine-5-dUTP (Roche), respectively. Digoxigenin detection was made using anti-digoxigenin-fluorescein antibody (Roche).

### Chromosome preparation and in situ hybridization

Germination, metaphase accumulation, and fixation procedures were carried out according to Kwiatek et al. (2016a). The chromosome preparations were made according to Hasterok et al. (2006). FISH and GISH experiments were performed for the identification of chromatin introgression. The analysis of genomic composition of hybrid plants was carried on the mitotic chromosomes of root meristems. Four probes (5S rDNA, 25S rDNA, pSc119.2, and pAs1) were subjected to in situ hybridization on the same chromosome preparations. First FISH was made according to Książczyk et al. (2011) with minor modifications of Kwiatek et al. (2013) using 5S rDNA (pTa794) and 25S rDNA (used for the detection of 25-5.8-18S rDNA loci). The hybridization mixture (40 μl per slide) contained 90 ng of each probe in the presence of salmon sperm DNA, 50 % formamide, 2 × SSC, and 10 % dextran sulphate, and was denatured at 75 °C for 10 min and stored on ice for 5 min. Chromosomal DNA was denatured in the presence of the hybridization mixture at 75 °C for 5 min on a heating table (Medax) and allowed to hybridize overnight at 37 °C. Digoxigenin detection was made using anti-digoxigenin-fluorescein antibody (Roche). After documentation of the FISH sites, the slides were washed according to procedure of Heslop-Harrison (2000) (2 × 45 min in 4 × SSC Tween and 2 × 5 min in 2 × SSC, at room temperature). Second FISH with pSc119.2 and pAs1 was made with the same conditions after reprobing followed by GISH carried out according to Kwiatek et al. (2012) with modifications. GISH experiments were performed using D-genome probe (from *Ae. tauschii*) and unlabelled triticale genomic DNA which was used as specific blocker. Multicolour GISH (mcGISH) was carried out using A-genome probe (from *T. monococcum*), D-genome probe (from *Ae. tauschii*) as well as unlabelled *Ae. speltoides* and *S. cereale* genomic DNA which were used as specific blockers. The GISH mixture (40 μL per slide), containing 50 % formamide, 2 × SSC, 10 % dextran sulphate, 90 ng each of the genome probes, and 4.5 μg blocking DNA, was denatured at 75 °C for 10 min and stored on ice for 10 min. The chromosomal DNA denaturation, hybridization, and immunodetection conditions were the same as in FISH experiments. Mitotic cells were examined with an Olympus BX 61 automatic epifluorescence microscope with Olympus XM10 CCD camera. Image processing was carried out using the Olympus Cell-F (version 3.1; Olympus Soft Imaging Solutions GmbH: Münster, Germany) imaging software and PaintShop Pro X5 software (version 15.0.0.183; Corel Corporation, Ottawa, Canada). The identification of particular chromosomes was made by comparing the signal pattern of selected probes (5S rDNA, 25S rDNA, pSc119.2, and pAs1) according to a previous study (Kwiatek et al. 2013, 2015) and similar cytogenetic analysis (Cuadrado and Jouve 1994; Schneider et al. 2003, 2005; Wiśniewska et al. 2013).

### PCR amplification of SSR markers

Genomic DNA was extracted from fresh leaves of single plants using GeneMATRIX Plant and Funghi DNA Purification Kit (EURx Ltd.). Total genomic DNAs of *Ae*. *tauschii*, ×*Triticosecale* cv. Bogo, *T. aestivum* cv. Chinese Spring, BC_2_F_5,_ and BC_2_F_6_ hybrids were used as templates for PCR. Analyses were performed with 25 μl mixture according to Kwiatek et al. (2016a). PCR reactions were carried out in LabCycler thermocycler (SensoQuest Biomedizinische Elektronik, Goettingen, Germany) according to programs reported in GrainGenes 2.0 Database (http://wheat.pw.usda.gov/GG2/index.shtml) for every SSR marker. Amplification products were electrophoresed at 120 V for about 2 h in 2 % agarose gel (Sigma), stained with ethidium bromide (Sigma), visualized under UV light and photographed (Syngen UV visualiser).

### Analysis of plant height, spike morphology, and effectiveness of self-pollinations

Measurements of plants height were performed when plants reached maturity. There were measured the length of stem and spike separately for every stem of the plants (1–8), and the mean values were calculated. For every plant, the spike morphology was evaluated and all the spikes were archived by photography. After the harvest, grains from spikes were threshed and the mean effectiveness of sell-pollinations for every plant was calculated. The number of obtained seeds was divided by the number of flowers in spike and expressed as a percentage value. For triticale cv. Bogo, ten representative plants were subjected to analysis regarding all mentioned phenotypic features.

### Evaluation of powdery mildew infections

During the vegetation period, the level of powdery mildew natural infection was evaluated according to COBORU (the Research Centre for Cultivar Testing) recommendations on a 9° scale, where 9 is the most favourable state for agriculture. The means of powdery mildew expression scores in BC_2_F_5_ and BC_2_F_6_ hybrids, *Ae*. *tauschii*, and triticale ‘Bogo’ were compared each year to the results of PCR amplification of *Pm43* marker using ANOVA calculations and Tukey’s HSD test.

## Results

### Identification of *Ae. tauschii* chromatin introgression in triticale hybrids

**Fig. 1:**
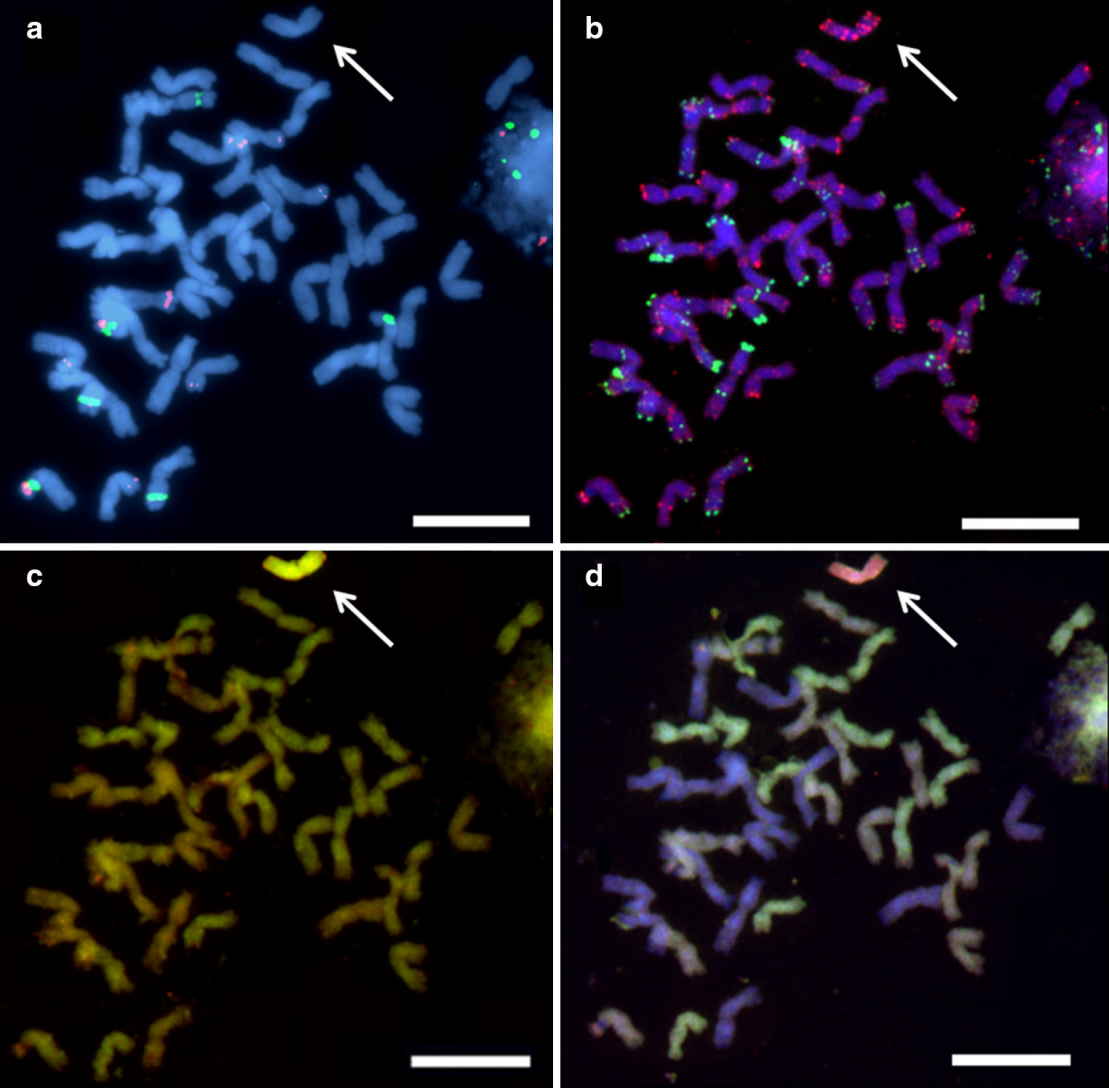
Mitotic chromosomes of BC_2_F_5_ (*Ae*. *tauschii* × *S*. *cereale*) × *Triticosecale* cv. Bogo hybrid analyzed using **a** FISH with 5S rDNA (*red*) and 35S rDNA (*green*) probes, **b** FISH with pAs1 (*red*) and pSc119.2 (*green*) probes, **c** GISH with total genomic DNA probes of *Ae*. *tauschii* (D, *green*) and triticale (ABR, *orange*), **d** multicolor GISH with total genomic DNA probes of *T*. *monococcum* (A, *green*), *S*. *cereale* and *Ae*. *speltoides* (R and B, *blue*), and *Ae*. *tauschii* (D, *red*). *Arrows* indicate the introgressed chromosome 2D. *Scale bar* 10 µm (color figure online)

**Fig. 2:**
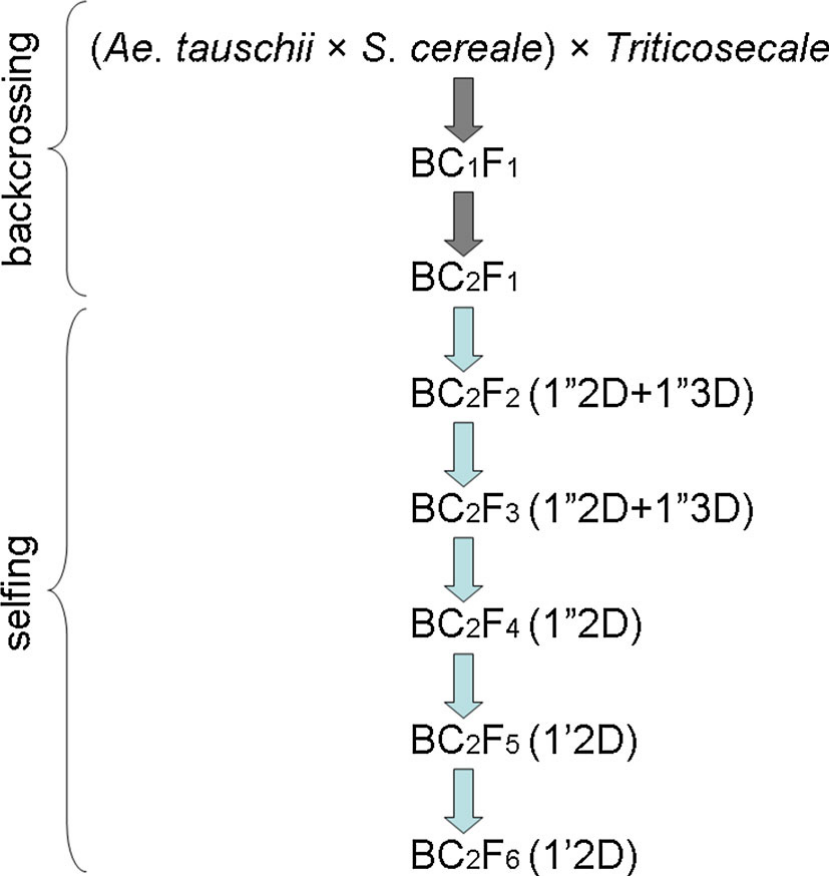
Scheme of subsequent crosses between *Aegilops tauschii* × *Secale cereale* amphiploid forms and triticale cv. Bogo. The presence of D-genome chromosomes in subsequent generations was in *brackets*

**Fig. 3:**
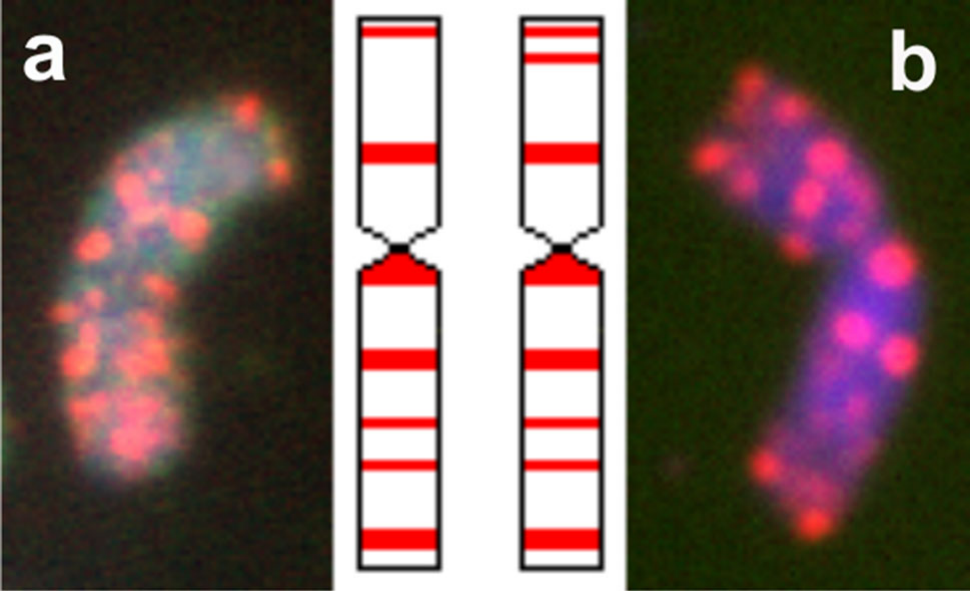
Idiogram and karyotype of two types of additional chromosomes of *Ae*. *tauschii* detected in the background of BC_2_F_5_ and BC_2_F_6_ hybrids of triticale showing genomic distribution of repetitive DNA sequence pAs1 (*red*). **a** Normal chromosome 2D, **b** chromosome 2D with rearrangement (color figure online)

The chromosome composition of (*Ae*. *tauschii* × *S*. *cereale*) × triticale cv. Bogo hybrids was performed using FISH and GISH. The analysis were made using FISH with 5S and 25S rDNA (Fig. 1a), pSc119.2 and pAs1 (Fig. 1b), GISH with D-genome probe, and blocking DNA from triticale (Fig. 1c), as well as mcGISH with A- and D-genome probes and blocking DNA from *Ae*. *speltoides* and rye (Fig. 1d). Identification of particular chromosomes of A-, B-, R-, and D-genome was made basing on previous reports of Cuadrado and Jouve (2002), Schneider et al. (2003, 2005) and Molnár et al. (2014), respectively, and chromosome arms ratio. The analysis of F_1_ to BC_2_F_3_ generations was performed by Kwiatek et al. (2015) and revealed that one hybrid plant from BC_2_F_2_ exhibited 46 chromosomes with additional pairs of 2D and 3D chromosomes. This plant was self-crossed to produce BC_2_F_3_ generation. The analysis of the progeny revealed six plants with additional pairs of 2D and 3D chromosomes as well as four plants with the addition of single chromosomes 2D and four plants with single chromosome 3D. The BC_2_F_4_ to BC_2_F_6_ plants in this study were obtained only from BC_2_F_3_ genotypes characterized by constitution of 21” + 1”3D + 1”2D chromosomes by subsequent self-pollinations (Fig. 2). FISH experiments allowed to distinguish 1 plant of BC_2_F_4_ with additional pair of 2D chromosomes. Eight plants of BC_2_F_5_ generation consisted of five plants with introgression of single chromosome 2D. Another three plants carried one rearranged chromosome 2D considering the differences in pAs1 sequence signals pattern (Fig. 3). Furthermore, 36 plants of BC_2_F_6_ generation were characterized by the presence of individual chromosome 2 of D-genome chromatin. Among BC_2_F_6_ hybrid plants, twenty carried single chromosome 2D, whereas sixteen carried one rearranged chromosome 2D.

### SSR markers analysis specific for D-genome chromatin

**Fig. 4:**
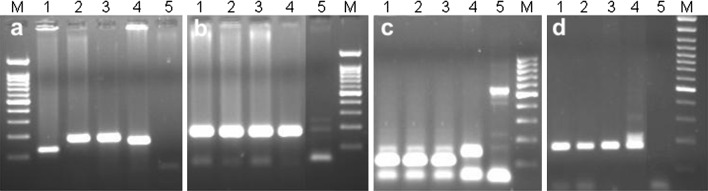
Molecular analysis of selected SSRs specific for chromosome 2D in genomes of hybrid plants with a rearranged chromosome 2D; **a** *Xgwm261*, **b** *Xgwm301*, **c** *Xgwm539*, **d** *Xgwm210*; hybrid plants with rearranged 2D chromosome (*1*), hybrid plants with whole 2D chromosome (*2*), *Ae*. *tauschii* (*3*), *T*. *aestivum* cv. Chinese Spring (*4*) and triticale cv. Bogo (*5*). Marker (*M*) size 100 bp

The analysis of molecular markers, specific for every D-genome chromosome according to wheat (*T*. *aestivum* cv. Chinese Spring) genetic maps reported by Somers et al. (2004) (*Xcfd19*-1D; *Xgwm301*-2D; *Xbarc71*-3D; *Xcfd71*-4D; *Xcfd10*-5D; *Xcfd49*-6D; *Xgwm428*-7D) confirmed the FISH/GISH results. The presence of 2D chromosome in eight plants of BC_2_F_5_ generation was confirmed in comparison with positive controls, which consists of *Ae. tauschii* and negative control of × *Triticosecale* cv. Bogo (Fig. 4). Furthermore, the organization of this chromosome was analyzed using 13 SSR markers (*Xcfd56*-2DS; *Xgdm35*-2DS; *Xcfd51*-2DS; *Xwmc25*-2DS; *Xwmc503*-2DS; *Xgwm261*-2DS; *Xgwm296*-2DS; *Xgwm210*-2DS; *Xgwm455*-2DS; *Xgwm102*-2DS; *Xgwm157*-2DL; *Xgwm539*-2DL; *Xgwm301*-2DL) with special consideration of the short arm, where *Rht8* is located. SSR markers’ analysis provided polymorphic sizes of bands considering three hybrid plants of BC_2_F_5_ generation. It was revealed that the place of rearrangement occurred between markers *Xcfd51* and *Xgwm210* in the short arm of 2D chromosome. The highest polymorphism was observed considering the sizes of amplification products of *Xgwm261* marker which is linked to *Rht8* gene (Fig. 4). The occurrence of the rearrangement in this region was also observed in plants of the next generation (BC_2_F_6_). It is worth to mention that in the case of most SSR markers, the size of the bands differs between selected *Ae*. *tauschii* line D51 and *T*. *aestivum* cv. Chinese Spring, however, appropriate bands were not present in triticale cv. Bogo. Weak bands appeared in negative control considering *Xgwm539*, *Xgwm301*, *Xgdm35*, and *Xgwm157* markers, which were detected in hybrid plants, as well.

### Evaluation of the influence of 2D chromosome addition on plant height and spike morphology

**Fig. 5:**
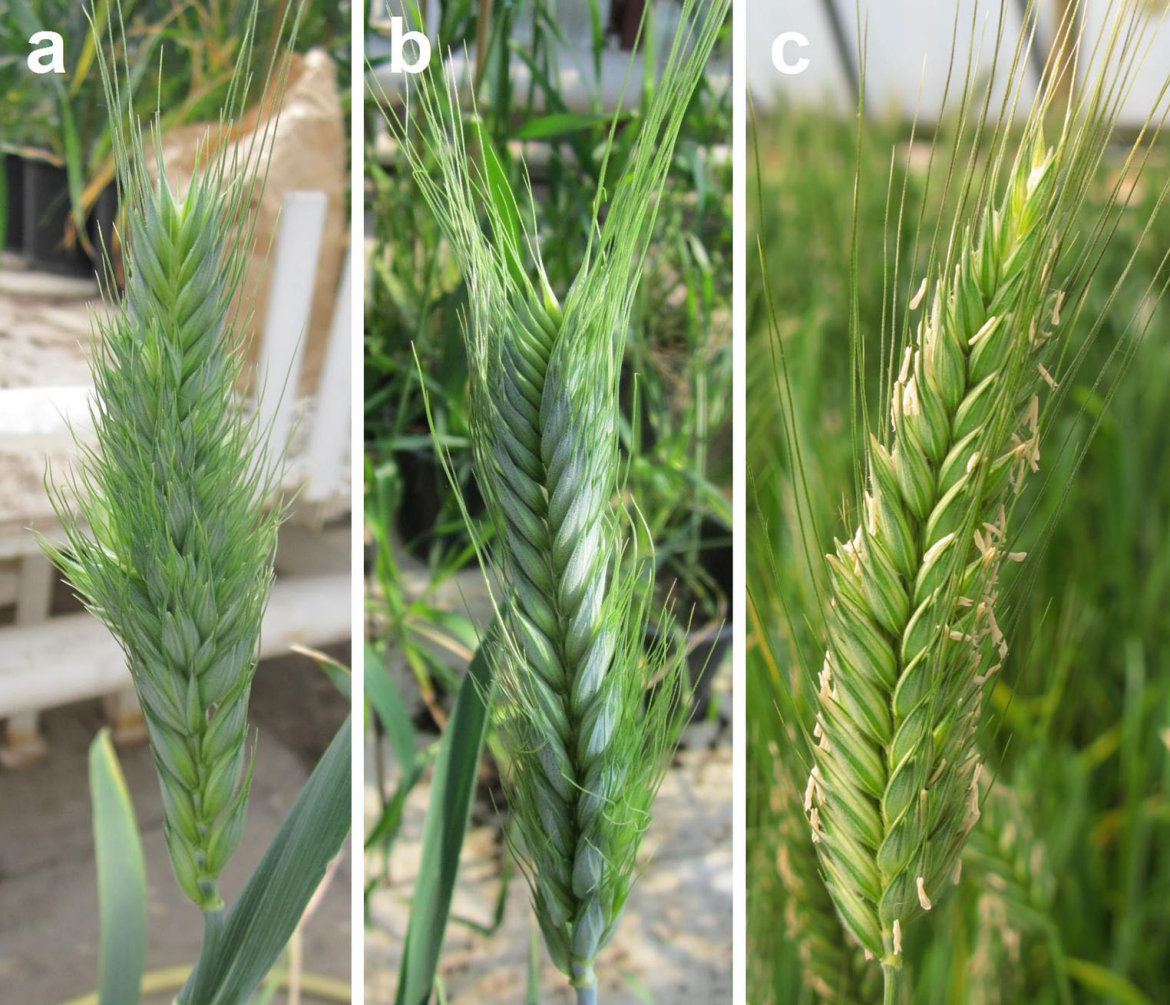
Spikes morphology of the analyzed plants. Spikes of the BC_2_F_6_ hybrids (*Ae*. *tauschii* × *S*. *cereale*) × *Triticosecale* cv. Bogo demonstrating **a** the presence of supernumerary spikelets and **b** morphology similar to triticale; **c** appropriate spike of the triticale cv. Bogo

In general, all monosomic alien addition plants of BC_2_F_5_ generation were lower in comparison with triticale (Table 1). The mean height of hybrid plants was 68 cm, whereas triticale plants were 26 cm higher (94 cm) what indicates about 28 % of height reduction. All hybrid plants of BC_2_F_5_ were treated as one group due to the small number of plants with D-genome introgression. In turn, all BC_2_F_6_ hybrids (36 plants) were divided into two groups: plants with chromosome 2D and plants which rearranged chromosome 2D according to the results of cytogenetic and SSR markers analysis. This observation revealed that plants with chromosome 2D and rearranged chromosome 2D lead to the decrease of plant height of about 37 (39 %) and 12 cm (12.5 %), respectively, in comparison with triticale (96 cm). When all hybrid plants of BC_2_F_6_ were treated as one group, such as in previous season, the mean value and reduction of plant heights were similar in both years (data not shown). The spike morphology was normal among BC_2_F_5_ hybrid plants in comparison with triticale cv. Bogo. In contrast, spikes of 16 hybrid plants of the next generation (22 % of all hybrids of BC_2_F_6_ generation) were characterized by the presence of supernumerary spikelets (SS) generally in the lower third of spike. However, there are also three plants which can be characterized by many additional spikelets along the whole length of the spikes (Fig. 5a). This trait in BC_2_F_6_ generation of hybrids leads to the increased fertility (64 %) in comparison with hybrid plants demonstrating spikes similar to normal triticale (46 %) (Fig. 5b), whereas triticale cv. Bogo (Fig. 5c) revealed high fertility (90 %) (Table 2). Comparing the mean fertility values for both generations of hybrids, the obtained results were similar and indicate about 44 % reduction of grains yield in comparison with triticale. Analysis with *Xgwm102* marker localized nearby *mrs1* in bread wheat revealed that the amplification product of 150 bp size was present in all analyzed hybrid plants and *Ae*. *tauschii* and was not present in triticale cv. Bogo.

**Table 1:** Analysis of BC_2_F_5_, BC_2_F_6,_ and parental form triticale cv. Bogo plant height

Generation	Number of plants	Mean height (cm)	Range of plants height (cm)	Mean reduction of height (cm)
BC_2_F_5_ (2D addition lines)	8	68	46–92	26
BC_2_F_5_ (without D chromatin)	21	88	79–96	6
Triticale cv. Bogo	20	94	86–101	N/A
BC_2_F_6_ (whole chromosome 2D)	20	59	43–70	37
BC_2_F_6_ (2D with rearrangement)	16	84	68–98	12
BC_2_F_6_ (without D chromatin)	36	90	83–101	6
Triticale cv. Bogo	20	96	89–104	N/A

The table represents mean height, range of plant height, and the observed reduction of height in the subsequent generation of plants

**Table 2:** Analysis of BC_2_F_5_, BC_2_F_6,_ and parental form triticale cv. Bogo fertility

Generation	Number of plants	Self-pollination effectiveness (%)	Range (%)
BC_2_F_5_ (2D addition lines)	8	42	15–81
BC_2_F_5_ (without D chromatin)	21	70	50–81
Triticale cv. Bogo	20	86	80–93
BC_2_F_6_ (all spikes)	72	46	17–83
BC_2_F_6_ (spikes with SS)	16	64	41–83
Triticale cv. Bogo	20	90	81–95

The table represents the mean and range of the effectiveness of self-pollinations in the subsequent generation of plants

### Evaluation of expression of the resistance to powdery mildew provided by *Pm43* gene from *Ae. tauschii* in triticale monosomic addition plants carrying 2D chromosome

During the vegetation period, all analyzed plants of *Ae*. *tauschii* exhibited low level of powdery mildew natural infection and mean scores ranged between 8.3 and 8.45 (Table 3). The observations of the infection symptoms conducted on all analyzed monosomic addition plants of BC_2_F_5_ and BC_2_F_6_ generations showed high tolerance to powdery mildew. There were no differences in resistance between hybrids carrying normal 2D and rearranged 2D chromosome. The mean scores of infection ranged between 7.63 and 7.97. In comparison, triticale cv. Bogo plants were less tolerant and the mean scores of the infection level ranged between 2.85 and 3.05 (Table 3). This observation was in accordance with the results of the SSR markers analysis with *Xgwm539* marker, located on chromosome 2D of *Ae. tauschii*, which is one of the markers related to powdery mildew resistant gene *Pm43* (Fig. 4). The amplification products of 150 bp in size were found in DNA extracts of *Ae*. *tauschii* (positive control carrying *Pm43*) and all monosomic addition plants of BC_2_F_5_ and BC_2_F_6_ generations. There were no differences in resistance between hybrids carrying normal 2D and rearranged 2D chromosome. The marker for *Pm43* was not identified in triticale ‘Bogo’ (negative control).

**Table 3:** Evaluation of the natural infection level caused by *B*. *graminis* on the BC_2_F_5_ and BC_2_F_6_ hybrids of (*Ae*. *tauschii* × *S*. *cereale*) × triticale ‘Bogo’

Generation	Number of plants			
	With *Pm43* marker		Without *Pm43* marker	
	*Ae*. *tauschii*	Hybrids	Triticale ‘Bogo’	Hybrids
BC_2_F_5_	20	8	20	21
BC_2_F_6_	20	36	20	36

Generation	Means (range) of infection scores			
	With *Pm43* marker		Without *Pm43* marker	
	*Ae*. *tauschii* (1)	Hybrids (2)	Triticale ‘Bogo’ (3)	Hybrids (4)
BC_2_F_5_	8.3 (8–9)	7.63 (7–9)	2.85 (2–4)	3.33 (2–4)
BC_2_F_6_	8.45 (8–9)	7.97 (7–9)	3.05 (2–4)	3.44 (2–4)

Generation	HSD level		1 vs 2	1 vs 3	1 vs 4	2 vs 3	2 vs 4	3 vs 4
	HSD_0.05_	HSD_0.01_						
Tukey’s honest significant difference (HSD) test								
BC_2_F_5_	0.63	0.77	*P* < 0.05	*P* < 0.01	*P* < 0.01	*P* < 0.01	*P* < 0.01	NS
BC_2_F_6_	0.46	0.57	*P* < 0.05	*P* < 0.01	*P* < 0.01	*P* < 0.01	*P* < 0.01	NS

## Discussion

The two groups of chromosomes within Triticeae tribe are considered to posses high density of genes (Conley et al. 2004). According to the literature, chromosome 2D of *Aegilops tauschii* especially carries the *Rht8* gene which determine semi-dwarfism feature and *Pm43* gene related to the resistance to *Blumeria graminis* (Jia et al. 2013). Furthermore, this chromosome bears gene *mrs1* responsible for SS (supernumerary spikelets) trait in bread wheat (Dobrovolskaya et al. 2015). In this work, we investigate the influence of the 2D chromosome of *Ae. tauschii* in monosomic addition plants of triticale considering agronomically important traits.

One of our assumptions was to introduce *Rht8* gene determining semi-dwarfism in wheat to triticale using *Ae. tauschii* monosomic addition lines, which will be employed for 2R/2D substitution or translocation induction. In our work, we used *Ae*. *tauschii* × *S*. *cereale* amphiploids as a bridge forms for the introgression of D-genome chromatin to triticale. Such a comprehensive approach was applied by Kwiatek et al. (2016a), who transfer the *Pm13* gene from *Ae*. *variabilis* × *S*. *cereale* amphiploids into triticale cv. Lamberto. The present results show that introgression of 2D chromosomes, bearing *Rht8* gene, from *Ae. tauschii* into triticale chromosome complement was successful and led to the height reduction of monosomic alien addition plants. Furthermore, this feature was maintained in the subsequent generations of hybrid plants. All monosomic alien addition plants, namely, 8 of BC_2_F_5_ and 36 plants of BC_2_F_6,_ for which PCR amplification revealed bands for *Xgwm261* marker, were substantially lower than triticale. The presence of the *Ae. tauschii* type band (200 bp) in hybrids can be associated with the reduction of height of about 37 cm in comparison with triticale cv. Bogo which mean height was 96 cm. Similarly, 16 hybrids in which rearrangement event took place were also lower than triticale; however, the mean reduction of height was 12 cm only (Table 1). The presence of *Rht8* in wheat leads up to 10 cm reduction of the European varieties (Worland et al. 1998); however, *T*. *aestivum* is lower than triticale generally because of the presence of the remaining semi-dwarfism genes. The absence of these genes with the presence of the “alien” semi-dwarfism gene *Rht8* in winter triticale is most likely the general reason of such high decreasing of plants height. Furthermore, our analysis revealed differences in size of amplification products of *Xgwm261* marker between positive controls—*Ae*. *tauschii* and *T*. *aestivum* cv. Chinese Spring. The amplification products of 200 bp in size were characteristic to *Ae. tauschii* and monosomic alien addition plants of BC_2_F_5_ and BC_2_F_6_ generations. SSR analysis of hybrid plants carrying rearranged 2D chromosome revealed amplification products of about 140 bp. Band of predicted size 192 bp was obtained for DNA of positive control ‘Chinese Spring’. The marker for *Rht8* was not identified in triticale cv. Bogo. That observations indicate that *Ae*. *tauschii* posses different alleles of *Rht8* gene, where wheat ‘Chinese Spring’ can be characterized by the most frequent allele *Rht8c*. According to McIntosh et al. (2003) analyzed *Ae*. *tauschii* accession might posses allele *Rht8d* (201 bp) because of the similarity of amplification product size.

Monosomic addition lines of triticale carrying chromosome 2D were also investigated in terms of powdery mildew resistance identification. According to Jia et al. (2013), a powdery mildew resistance gene *Pm43* was mapped on the 2D chromosome of wheat. The molecular marker analysis and the visual evaluation of powdery mildew symptoms in *Ae*. *tauschii* revealed the presence of *Pm43* marker (*Xgwm539*) and low powdery mildew reaction, confirmed by infection scores made on 20 plants each year of the experiment (Table 3). Similarly, monosomic alien addition plants of the BC_2_F_5_ and BC_2_F_6_ generations were highly tolerant to powdery mildew infection and possessed *Pm43* marker. A lack of the differences in resistance between hybrids with additional 2D and rearranged 2D chromosome is related to the localization of the *Pm43* marker on the long arm of chromosome 2D. In comparison, triticale ‘Bogo’ was much more infected, which was confirmed by Tukey’s HSD test (Table 3). It is in accordance with the results of Czembor et al. (2013) who reported that triticale ‘Bogo’ is susceptible to all isolates of *Blumeria graminis* derived from triticale. Furthermore, the molecular analysis showed that *Pm43* marker was not present in triticale ‘Bogo’ (Fig. 4; Table 3). *Pm43* gene was mapped on the basis of CH5025 × CH5065 2DL population source map (Jia et al. 2013). CH5025 was a *Th. intermedium*-derived line of wheat resistant to powdery mildew, whereas CH5065 is susceptible line (He et al. 2009). On this basis, it can be assumed that the region determining resistance to powdery mildew in *Th*. *intermedium* is homologous in *Ae*. *tauschii* what is consistent with observations of He et al. (2009) about chromosome pairing of D-genome of wheat and J or J^s^ chromosomes of *Th*. *intermedium*. Furthermore, in both the species as well as in obtained hybrids, the resistance was maintained at both the stages of development. The successful transfer of 2D chromosomes and positive effect of powdery mildew resistance in triticale proved that *Ae*. *tauschii* is more valuable source of this gene for breeding programs because of the simplest genome composition and closer relationship with traditional crops, rather than *Th*. *intermedium*.

Our work revealed a rearrangement of chromosome considering additional chromosome 2D in the subsequent generations of triticale plants. The chromosome aberrations appeared between markers *Xcfd51* and *Xgwm210* in the short arm of chromosome 2D. This region is notably important forasmuch, and there is localized semi-dwarfing gene *Rht8*. The changes in organization of 2D chromosome had direct influence on plant height of triticale hybrids, what might be connected with changes in *Rht8* loci which is localized nearby *Xgwm261*. Although the occurrence of rearrangement was unexpected in such advance generations of plants, its localization in chromosome is not random and might be explained by the evolution of *Ae*. *tauschii* genome. According to the literature, based on the sequencing data, it was hypothesized that the seven chromosomes of this species originated from 12 ancestral chromosomes by five nested chromosome insertions (NCIs). During the NCIs, a telomere of the inserted chromosome was inserted near the centromere in a gene-containing region. As a result of NCIs, one of the centromere was lost, and the centromere of the inserted chromosome became the active centromere in each compound chromosome. Chromosome 2D of *Ae*. *tauschii* was produced as a result of the NCIs. Therefore, it might be concluded that examined in this research region is specially exposed to chromosome rearrangements and such places in genome may constitute a hot spots. Furthermore, the heat map of the recombination rates showed that the recombination rate for this region is relatively high (Luo et al. 2009, 2013). That might be the reason of rearrangement events which occur especially during stress conditions, such as integration of the alien chromatin into hybrid plants or backcrossing.

The results of this research showed that the presence of additional chromosomes 2D induces changes in spike morphology of triticale hybrid plants. However, measurements of spikes revealed any significant differences in the length of the spike in hybrid plants (data not shown). Most of the analyzed plants were characterized by the presence of spikes which morphology was congenial to triticale spike what should be expected in advanced generations of hybrids (Fig. 5b). However, about 22 % of BC_2_F_6_ generation plants posses spikes with supernumerary spikelets, a trait which was already reported only for wheat, where the MRS trait is under the control of a recessive allele at a single locus located on chromosome 2D (Dobrovolskaya et al. 2015). The presence of *bh*-*D1*, a multirow spike recessive allele (alias *mrs1*), was also reported for *Ae*. *tauschii* (Jia et al. 2013). SSR marker analysis with *Xgwm102* marker revealed amplification products of 150 bp in size for *Ae*. *tauschii* and unexpectedly for all hybrid plants. Considering the obtained results and wheat origin of this SSR marker (genetic distance 1.3 cM), it was assumed that this marker is not suitable to analyze the SS trait in hybrid plants of *Ae*. *tauschii* and triticale. This relevance proves that the genetic distance between *bh*-*D1* and *Xgwm102* marker on map reported by Jia et al. (2013) is 9.43 cM which indicates that this markers in not combined with SS trait in *Ae*. *tauschii*. It is also important that this recessive trait in triticale hybrids was conditioned only by the presence of single chromosome 2D which is consistent with the observations of Sears (1954), who report the reduplication of spikelets in hexaploid wheat plants nullisomic for this chromosomes. The occurrence of SS trait in analyzed plants leads to the increase of grain yield in comparison with the rest pool of plants (Table 2). These observations allowed to conclude that such a approach may allow new spike architectures of triticale to be designed, with the aim of enhancing grain production.

In conclusion, monosomic alien addition plants obtained here provide a feasible platform to identify and estimate valuable traits for triticale breeding using molecular cytogenetics, screening of forms with SSR markers combined with phenotype analysis, and evaluation of powdery mildew infection. Using these methods, we have obtained 20 semi-dwarf plants of BC_2_F_6_ generation carrying 2D chromosome with the powdery mildew resistance, without changes in spike morphology which can be used in the triticale breeding programs. Furthermore, the limitations of reports about transferring the powdery mildew resistance genes as well as genes determining semi-dwarfism and spike morphology to triticale indicate that there is a great legitimacy and potential in the field of using monosomic addition plants carrying chromosomes of *Ae*. *tauschii* to improve this valuable crop. From the other hand, the molecular analysis of triticale monosomic alien addition plants provided significant insights considering the organization changes of additional chromosome induced by the integration of the alien chromatin in triticale genetic background.
